# Assessment of coastal sustainable development along the maritime silk road using an integrated natural-economic-social (NES) ecosystem

**DOI:** 10.1016/j.heliyon.2023.e17440

**Published:** 2023-06-19

**Authors:** Jian Zuo, Li Zhang, Bowei Chen, Jingjuan Liao, Mazlan Hashim, Dewayany Sutrisno, Mohammad Emran Hasan, Riffat Mahmood, Dalhatu Aliyu Sani

**Affiliations:** a Key Laboratory of Digital Earth Science, Aerospace Information Research Institute, Chinese Academy of Sciences, Beijing, 100094, China; b International Research Center of Big Data for Sustainable Development Goals, Beijing, 100094, China; c University of Chinese Academy of Sciences, Beijing, 100049, China; d Geoscience & Digital Earth Centre (INSTEG), Research Institute for Sustainable Environment (RISE), Universiti Teknologi Malaysia, Johor Bahru, Malaysia; e Universiti Teknologi Malaysia, Johor Bahru, Malaysia; f Center for Research, Promotion and Cooperation, Geospatial Information Agency (BIG), Cibinong, 16911, Indonesia; g Climate Justice and Natural Resource Rights, Oxfam GB in Bangladesh, Mohakhali, Dhaka, 1206, Bangladesh; h Department of Geography and Environment, Jagannath University, Dhaka, 1100, Bangladesh; i Department of Geography, Yusuf Maitama Sule University, Kano, Nigeria

**Keywords:** Sustainable development, Coastal marine ecosystem, Natural-economic-social ecosystem, Maritime silk road

## Abstract

Understanding spatial change and its driving factors behind coastal development is essential for coastal management and restoration. There is an urgent need for quantitative assessments of sustainable development in the coastal ecosystems that are most affected by anthropogenic activities and climate change. This study built a theme-based evaluation methodology with the Natural-Economic-Social (NES) complex ecosystem and proposed an evaluation system of coastal sustainable development (CSD) to understand the complex interactions between coastal ecosystems and anthropogenic activities. The approach revealed the levels of coastal natural, economic, and social sustainable development in the countries along the Maritime Silk Road (MSR) from 2010 to 2020. The results showed (1) a decreasing trend for coastal sustainable development between 2010 and 2015 and a rapid increasing trend between 2015 and 2020; (2) spatially varied CSD, with higher levels in Europe and Southeast Asia and lower levels in South and West Asia and North Africa; and (3) a strong influence on CSD by a combination of economic and social factors and relatively little influence by natural factors. The study further assessed the natural, economic, and social development scores for 41 countries and compared them with the mean scores (MSR) to classify coastal development patterns into three stages (favorable, transitional, and unfavorable). Finally, in the context of the 2030 Agenda for Sustainable Development, the study highlighted the importance of more refined global indicators for CSD assessments.

## 1 Introduction

The coastal zone, the interface between land and sea, is one of the most dynamic, complicated, and frequently changing areas [1,[2], [3]]. Under the combined influence of climate change and human activities, the ecological environment of the coastal zones tends to deteriorate, and problems such as offshore eutrophication, biological invasion, ocean acidification and coastal wetland degradation have emerged, constantly threatening the coastal sustainable development [4]. Such ecological challenges of coastal zones stem from the complicated combination of socio-economic activities and ecological sustainability, which has emerged as a major source of concern in the United Nations study on sustainable development. With the proposal of the 17 Sustainable Development Goals (SDGs), especially SDG14, which aims to enhance the protection and sustainable management of coastal and marine ecosystems and their resources while addressing threats such as pollution and ocean acidification [5], there are new calls for scientific understanding of the issues involved in the sustainable development of coastal zones.

The Maritime Silk Road (MSR) is a commercial maritime route that aims to develop economic and cultural linkages between Southeast Asia, Oceania, the Indian Ocean, and East Africa [6,7]. It passes through the coastal zones of a large number of countries and involves a variety of climatic zones such as subtropical, tropical, and Mediterranean [8]. The coastal zones of MSR face several problems such as environmental pollution and ecological degradation caused by industrialization and urbanization, especially the vast majority of countries involved are developing countries and emerging economies [9]. Human activities exert pressure on coastal ecosystems in these areas, such as fishing, coastal reclamation, port transportation, and tourism [10], have had a significant effect on habitat loss, pollution, eutrophication, and species loss [11]. The MSR involves most types of coastal areas globally and faces the same global coastal problems. The exploration of its coastal sustainability can provide an important reference for global coastal sustainable development.

The concept of sustainable development is quite unclear and non-operational, particularly for complex coastal ecosystems [12]. It calls for supporting coastal livelihoods and driving economic growth based on ensuring marine ecosystems health, particularly in several key sectors [13]. These sectors include fisheries and aquaculture, tourism, energy, shipping and port activities, seabed mining, and innovative areas such as renewable energy and marine biotechnology. As a result, scientific knowledge is needed to integrate the concept of sustainable development with practical coastal development needs [14]. An assessment may address the demands of practical sustainable development implementation by producing a simplified and comprehensive depiction of the organization, structure, and function of complex ecological settings and their changes over time [15].

Quantitative measurement of the process or level of sustainable development is the basis of sustainable development research [16]. Since different researchers have different understandings of the system's objectives, as well as different evaluation methods and perspectives, the evaluation results are somewhat subjective; thus, scientific requirements for evaluation are needed [17]. For a conceptual framework that combines indicators, it is necessary to understand the system and the types of attributes and interactions that define it and then seek data that can be used to indicate these attributes [18]. Among them, scientific theoretical models and evaluation indicators are important tools for sustainable development assessment [19].

Commonly used theoretical models for coastal sustainability assessment include the ecological footprint model (EFM) [20], the ecological carrying capacity model (ECC) [21], and the comprehensive evaluation index system (CEIS) [22]. The CEIS has been used extensively in evaluating, addressing, and interchanging with respect to ecological issues worldwide, dominating with the Pressure-State-Response (PSR) model [23] and the Nature-Economy-Society (NES) model [24]. UN [25] indicated that comprehensive evaluation is helpful for integrating the three dimensions of sustainable development and can establish a connection between the root causes of ecological and environmental problems and the associated impacts of anthropogenic activities.

In addition to the model, an evaluation system is a vital tool for monitoring sustainable development . The DEDUCE (Sustainable Development of European Coastal Zones) supported by the Interreg III-South Community Initiative Programme gives a set of 27 core indicators consisting of 46 environmental economic, and social measurements to monitor the sustainable development of the coastal zone at different scales [26]. On the global scale, the Ocean Health Index (OHI) assesses the health of the ocean from the perspective of the coupled human-nature system by estimating the current and future state of ten different targets [27]. The OHI is flexible and can evaluate coastal health on a variety of scales. However, as the database used for the indicators of the OHI on coastal vitality and economy has not been updated since 2012, its assessment lacks economic and social support, resulting in an incomplete assessment system for coastal and marine ecosystems. Although many principles and theoretical frameworks have been proposed, a universal approach is still pending [28]. In particular, with the continuous development of big data technology and the promotion of the UN SDGs, various spatial data and statistics are being improved, and the interrelationships between the natural, economic and social dimensions are being explored [29]. There is an urgent need for a sustainable development assessment system that is fully based on various types of big data that better reflects coastal zone interactions.

The results of the current assessment of the level of coastal sustainability in countries along the MSR [[30], [31]] showed that more than half of the countries' coastal zone sustainability scores are below average in 2019, and there is still much room for improvement. In view of the MSR’s fragile ecological background and the severe shortage of relevant studies, it is necessary to further refine the indicators and evaluation models, which include looking at the spatial heterogeneity and temporal dynamics of ecosystems, modeling how ecological processes interact with human activities, and incorporating a variety of factors (climate change, human activities, ecological processes, etc.) .[32] To meet sustainable development assessment needs of the MSR, this study 1) proposed a coastal sustainable development (CSD) evaluation system based on the natural-economic-social (NES) model and a theme-based evaluation methodology to assess the sustainability of coastal development in the countries along the Maritime Silk Road; 2) analyzed the spatio-temporal pattern of the coastal sustainable development (2010–2020); and 3) investigated the key factors of coastal sustainable development and provided policy recommendations for countries along the MSR.

## 2 Materials and method

### 2.1 Study area

**Fig. 1:**
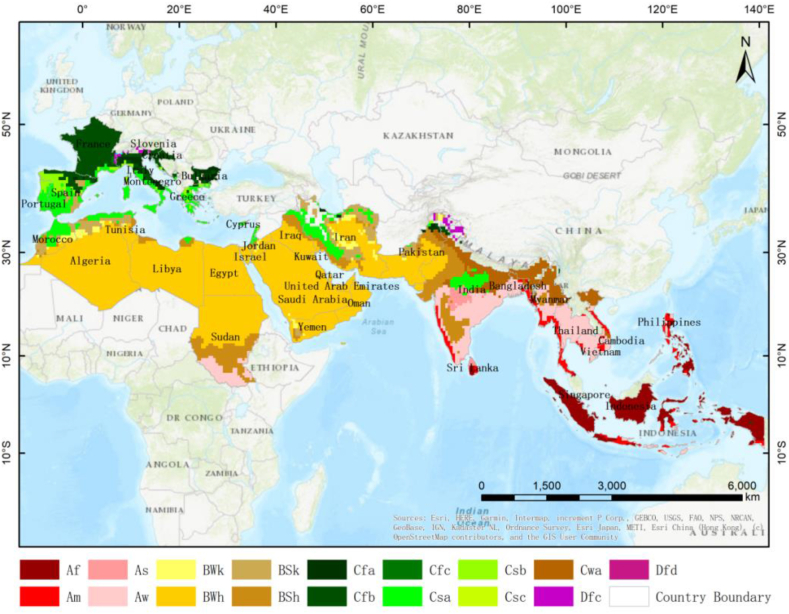
Study area: Countries along the Maritime Silk Road. The background map is the map of the Köppen-Geiger climate classification [39], where the main climates include A (equatorial), B (arid), C (warm temperate), and D (snow); the precipitation includes W (desert), S (steppe), f (fully humid), s (summer dry), w (winter dry), and m (monsoonal); the temperature includes h (hot arid), k (cold arid), a (hot summer), b (warm summer), c (cold summer), and d (extremely continental).

In our study, 41 countries along the MSR are considered (see Fig. 1). These countries are complex in natural environments with diverse and fragile ecological environments [33]. Most of them are developing countries, with a relatively crude mode of economic development, high energy and resource consumption, and low energy efficiency per unit [34]. Under the combined influence of climate change and human activities, the ecological environment of coastal zones in these regions tends to deteriorate [35,36]. Faced with the comprehensive and complex nature of the complex coastal ecosystems, it is essential to make a scientific and reasonable evaluation of the sustainable development capacity, so as to scientifically understand the problems in the process of sustainable development of the coastal zones, weigh the relationship between economic growth and environmental protection in the coastal zones, propose a sustainable future development path. This research defines the coastal zones as the intermediate area between the shoreline and 100 km inwards to the land [[[37], [38]]]. For small island countries, e.g., Singapore, a national scale is used for the coastal zones.

### 2.2 Evaluation system of coastal sustainable development level

**Fig. 2:**
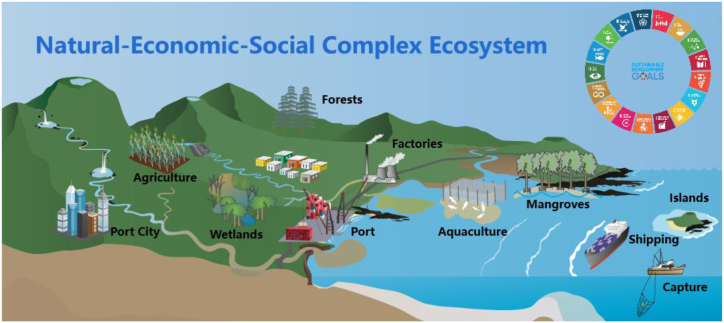
Natural-economic-social Complex Ecosystem for the coast. Symbols are courtesy of the Integration and Application Network, University of Maryland Center for Environmental Science (https://ian.umces.edu/symbols/).

The **evaluation system of the coastal sustainable development level** was built on the basis of the systematic classification framework of SDGs proposed by Ref. [40]; incorporating a theme-based evaluation methodology with the NES complex ecosystem (Fig. 2) as the theme. The Natural-Economic-Social (NES) complex ecosystem was shown in the appendix (Text S1) [41]. The evaluation system was referenced from the 2007 Sustainable Development Indicator Construction and Methodology (3rd edition) of the UN Commission on Sustainable Development (CSD) [42], the Ocean Health Index (OHI) [27], and the Coastal Sustainable Index of the nations along the MSR [30].

In our evaluation system, natural themes were divided into five sub-themes: atmosphere, coast, land, water, and biodiversity, culminating in a three-tier (theme - subtheme - indicators) assessment indicator system [43]. The system consisted of three themes, seven sub-themes, and 29 specific indicators (Table S1). It took full account of the coastal characteristics, covered the key issues of general national and regional concern such as ocean acidification and biodiversity conservation, and fully reflected the multidimensional needs of sustainable coastal development. It enabled this study to better represent the interconnected and multifaceted nature of sustainable development by assessing the three dimensions of sustainable development to better assist policy-making.

**Fig. 3:**
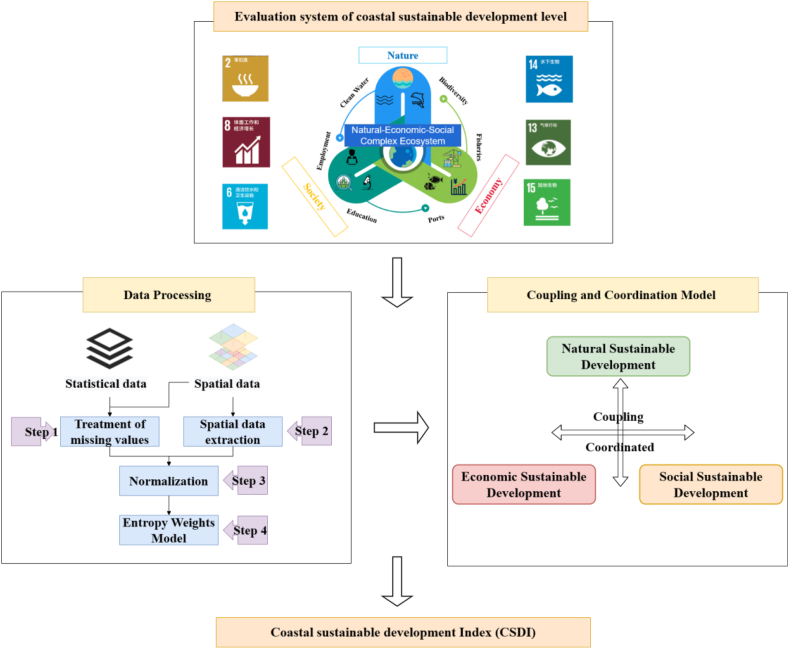
Technical flowchart for **evaluation system of coastal sustainable development level**.

Under the guidance of the above coastal sustainable development evaluation system, we collected the indicators data and imported big earth data to improve the construction of the indicators. The data processing process was shown in the appendix (Text S2) and the CSDI was obtained through the coupling and coordination model (Text S3) (see Fig. 3).

### 2.3 Spatial autocorrelation model

Spatial autocorrelation measures the nature and strength of interdependence among the values of a particular phenomenon over space (i.e., whether similar values tend to cluster together in space). To measure how CSDI is spatially autocorrelated among all the countries along the MSR, the Global Moran's I values were calculated and assessed with the null hypothesis [44]. The general forms of Moran's I are as Formula (1):

(1) $$Moran^{\prime }s\;I=\sum_{i=1}^{n}\sum_{j=1}^{n}W_{ij}\left(Y_{i}-\bar{Y}\right)\left(Y_{j}-\bar{Y}\right)/S^{2}\sum_{i=1}^{n}\sum_{j=1}^{n}W_{ij}$$

where n is the total number of countries; $W_{ij}$ is the spatial weight matrix to measure the distance between Country i and Country j; $S^{2}$ is the sample variance; $Y_{i}$ and $Y_{j}$ refer to Country i’ s and Country j’ s sample values, respectively; and $\bar{Y}$ is the sample average. The value range of Global Moran’s I is [−1,1]. When the value I is greater than 0, there is a positive correlation between spatial unit observations, and the greater the value is, the more significant the spatial correlation; when the value is lower than 0, there is a negative correlation, and the lower the value is, the more significant the spatial correlation; when the value is 0, the observed values are randomly distributed [45].

### 2.4 Geographical detector model for the main driving forces

The geographical detector (GeoDetector) model identifies interactions between several parameters using spatial superposition technology and set theory. The core hypothesis of GeoDetector is that if a significant spatial consistency exists between independent variable X and dependent variable Y, then the variables are correlated.

#### 2.4.1 Single factor detector

The factor detector identifies factors that are responsible for the independent variable. The explanatory power of each factor is measured by the q value as Formula (2):

(2) $$q=1-\frac{1}{N\sigma^{2}}\sum_{i=1}^{m}N_{i}\sigma_{i}^{2}$$

where q indicates the size of the contribution of Factor X to Factor Y and ranges from 0 to 1 (that is, 0 indicates no correlation between the two and 1 indicates that Y is completely dependent on X), N is the size of Y, $\sigma^{2}$ is the variance of variable Y, m is the number of layers, and $N_{i}$ and $\sigma_{i}^{2}$ represent the scale and the variance of the ith layer. The q value followed the noncentral F test [46], which was used to determine the significance level.

#### 2.4.2 Interaction detector

The interaction detector assesses whether the explanatory powers of two factors are enhanced, weakened, or independent of each other. First, the q values of two factors $X_{1}$ and $X_{2}$ for Y were calculated (${q(X}_{1})$ and ${q(X}_{2})$). Then, the q value of interaction, which is a new layer formed by the tangent of overlay variables $X_{1}$ and $X_{2}$, was calculated (${q(X}_{1}\cap X_{2})$) and compared with ${q(X}_{1})$ and ${q(X}_{2})$ to indicate the interaction type between two variables. According to the comparison results, it can be divided into five categories [47]:

(3) $${Independent\;of\;each\;other:q(X}_{1}\cap X_{2})=q\left(X_{1}\right)+q\left(X_{2}\right)$$

(4) $${Nonlinearly\;enhance\;each\;other:q(X}_{1}\cap X_{2})>{q(X}_{1}){+q(X}_{2})$$

Enhanceeachotherbidirectionally:Max

(5) $$\left({q(X}_{1}),{q(X}_{2})\right)<{q(X}_{1}\cap X_{2})<{q(X}_{1}){+q(X}_{2})$$

(6) $${Nonlinearly\;weaken\;each\;other:q(X}_{1}\cap X_{2})<\mathrm{Min}\left({q(X}_{1}\right),{q(X}_{2}))$$

(7) $${Weaken\;each\;other\;uniformly:\mathrm{Min}\left({q(X}_{1}\right),{q(X}_{2}))<q(X}_{1}\cap X_{2})\;<\mathrm{Max}\left({q(X}_{1}\right),{q(X}_{2}))$$

## 3 Results

### 3.1 Characteristics of the coastal sustainable development index (CSDI) for countries along the MSR

#### 3.1.1 Temporal characteristics of the CSDI in countries along the MSR

The study used the evaluation framework (Table S1), which was built based on natural-economic-social ecosystems, to assess the coastal sustainable development level of the countries along the MSR. The average score of the Coastal Sustainable Development Index (CSDI) for the countries along the MSR generally showed an increasing trend, from 42.8 in 2010 to 44.5 in 2020.

The three themes had varying degrees of volatility and increase, with the natural theme decreasing from 2010 (47.5) to 2015 (46.7) and then increasing to 2020 (49.3). The economic theme had a substantial increase from 2010 (19.0) to 2015 (21.0), followed by a smaller gain in 2020 (21.1), and the social theme had a considerable increase from 2010 (34.8) to 2015 (38.7), although the average score in 2020 (36.1) was lower than that in 2015. In terms of coupling coordination, a high level of coordination was maintained (∼50), with some degree of fluctuation but little overall change from 2015 (56.4) to 2020 (56.1), indicating that there was still space to strengthen the coordination of the three dimensions of sustainable development in the countries along the MSR.

**Fig. 4:**
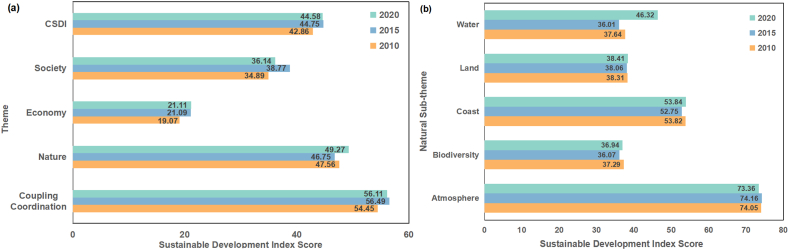
(a) The average of the coastal sustainable development index (CSDI), three main themes (nature-economy-society) and their coupling coordination scores for the countries along the MSR in 2010, 2015, and 2020; and (b) the average of five natural sub-themes (atmosphere, biodiversity, coast, land, and water) for the countries along the MSR in 2010, 2015, and 2020.

In terms of the difference between the largest and lowest scores, the difference between 2010 and 2015 decreased; however, the difference between 2015 and 2020 increased dramatically. The highest score for the sub-themes of the natural theme was for the atmosphere, although there was a drop in 2020 compared to the previous years. The remaining four sub-themes all showed a reduction from 2010 to 2015 but an increase from 2015 to 2020. The results showed the efficiency of recent efforts by nations in coastal habitat conservation under the guidance of SDG 14 [48]. Although the ratings for land resources and biodiversity both increased after 2015, the total score remained below 40, indicating a relatively low degree of sustainability with significant opportunity for improvement (see Fig. 4).

#### 3.1.2 Spatial characteristics of the CSDI in countries along the MSR

**Fig. 5:**
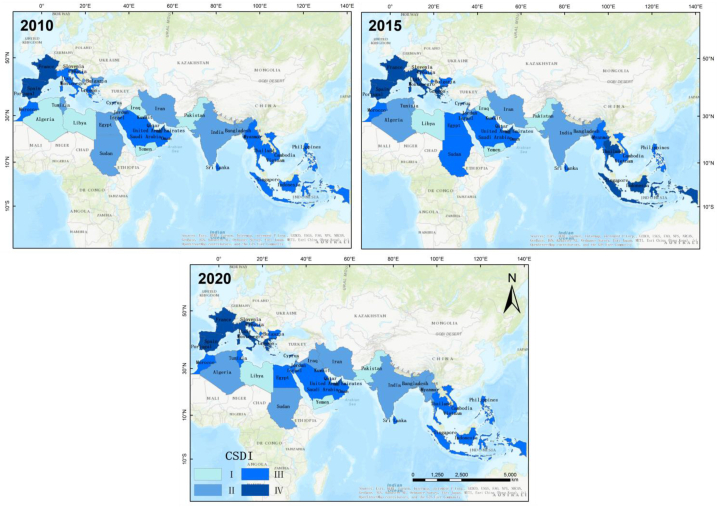
Spatial distribution of CSDI for the 41 countries along the MSR in 2010, 2015, and 2020.

To clearly show the spatial evolution of the sustainable development of the coastal zone along the MSR, the study divided the average value of the CSDI from 2010 to 2020 into four certain intervals according to the Jenks natural breaks classification. They were Ⅰ[28.0–34.5], Ⅱ[34.5–40.5], Ⅲ[40.5–50.5], and Ⅳ[50.5–60.5], respectively. The higher the CSDI score, the more sustainable the coastal complex ecosystem was. The geographical distributions in 2010, 2015, and 2020 were compared, and the overall CSDI of the MSR did not change much, but the changes in countries and regions were more obvious. The top 10 countries with a higher level of coastal sustainable development were concentrated in Europe and Southeast Asia (Ⅲ and Ⅳ). The countries with lower levels of sustainable development were concentrated in North Africa, South Asia, and West Asia, including Iraq, Yemen, Syria, Libya, Pakistan, Bangladesh, and Jordan (Ⅰ and Ⅱ). The countries consistently ranked at the top for the ten years included Portugal, France, Spain, Slovenia, Italy, and Greece in Europe; Singapore and Indonesia in Southeast Asia; and the United Arab Emirates in West Asia (see Fig. 5).

**Fig. 6:**
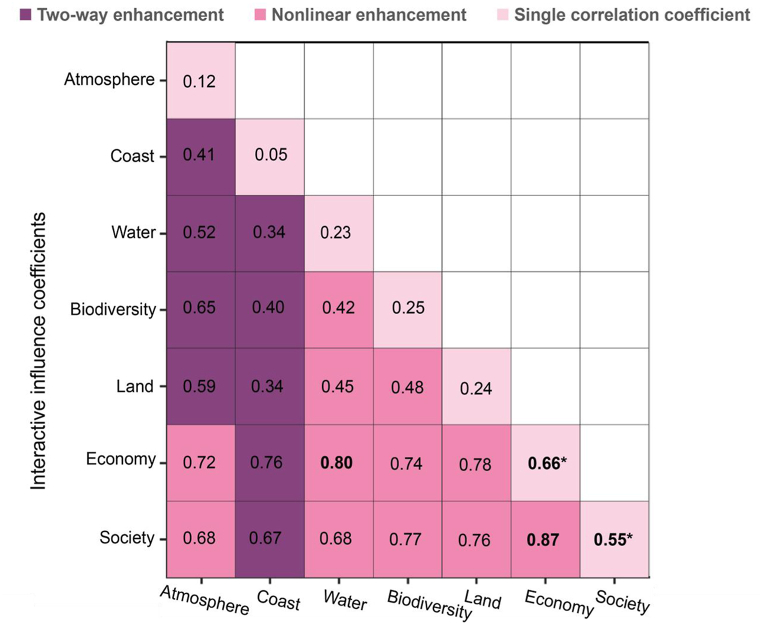
Interactive influence coefficients between socioeconomic themes and natural sub-themes of coastal regions. The correlation coefficient between CSDI and three main themes and natural sub-themes. *p value < 0.01.

Fig. 6 shows the single-factor geographic exploration and interaction detector between the factors, with a total of 2 themes (economy and society) passing the significance test to the CSDI (p < 0.01). The results of the interaction between socioeconomic themes and natural sub-themes of coastal regions had a significant two-way enhancement or nonlinear enhancement of CSDI. Economy and society had the highest explanatory power of 0.87, followed by the interaction of Economy and Water with an explanatory power of 0.80.

### 3.2 Spatial-temporal characteristics of the N-E-S themes and their coupling coordination

The top 10 countries in terms of natural development were in Southeast Asia (Brunei Darussalam, Indonesia, Myanmar, Cambodia, and the Philippines) and in Europe (Slovenia, Portugal, Spain, Croatia, and France). Most of these countries have relatively abundant coastal resources and depend on the sea for their livelihoods and have good coastal protection policies in place [49]. The European Union (EU), in particular, enacted the Commission Decision on the good environmental status of marine waters, which offered an overview of the extent to which good environmental status is reached in the EU's seas and oceans[50]. The bottom-ranking countries were mostly those in South Asia (Pakistan, Syria, India, and Bangladesh), West Asia (Lebanon, Israel, and Bahrain), and Africa (Libya and Tunisia). Most of these countries were still in the stage of higher intensity development of their coastal environment to support economic development, which placed more stress on the coastal environment [51]. The higher average natural sustainability scores were in Europe (natural score = 59) and Southeast Asia (natural score = 55), while the lower scores were in South Asia (natural score = 38). Countries with lower natural ratings often lack the resources and opportunities to address social or environmental issues, and they are unable to readily implement the governance changes required to alleviate social and environmental pressures [52].

While there was an upward trend in economic sustainability scores overall, the rate of growth from 2015 to 2020 was slower than that from 2010 to 2020. The top 10 countries in terms of economic development were from Europe (Spain, Portugal, Italy, France, and Greece) and Southeast Asia (Singapore, Philippines, and Thailand). Most of these countries were generally ranked high-income or middle-income countries according to the World Bank [53] standards. They not only had higher GDP per capita and GDP per capita growth rates, but also higher scores in port throughput and port infrastructure, a greater share of renewable resources for economic development, and better management of carbon emissions intensity [54], as shown in the scores of the economic indicators. The bottom-ranking countries were mostly from Africa (Libya and Algeria) and West Asia (Yemen, Iraq, and Syria). These countries were experiencing poor economic progress, notably in politically dangerous West Asian countries such as Iraq and Syria, which were classed as low-income countries by the World Bank [53]. However, according to the economic indicators, they ranked well in aquaculture and capture fisheries, especially African aquaculture output, which has experienced accelerated growth .

The top 10 countries in terms of social development were from Europe (France, Spain, and Portugal), Southeast Asia (Singapore), and West Asia (Qatar, the United Arab Emirates, and Israel). This finding was consistent with the United Nations Development Programme's Human Development Report 2020 (HDI), and the majority of these countries were more developed with greater levels of human development (UNDP, http://hdr.undp.org). They had a higher life expectancy and better employment opportunities and spent more in education and research, according to the social indicator scores. The countries at the bottom of the ranking were mainly South Asian countries (India, Sri Lanka, and Pakistan), which had a high score on population growth. Geopolitics, territorial conflicts, religious tensions, and other factors had a significant impact on them. The areas with higher average scores were Europe (social score = 43) and Southeast Asia (social score = 36); the regions with lower scores were South Asia (social score = 25). It should be noted that while the average for West Asia was high, there were significant differences across the area, with countries such as Qatar and the United Arab Emirates ranking higher and war-torn countries such as Iraq and Iran ranking lower.

The top 10 countries in terms of coupling coordination were from Europe (Portugal, France, and Spain) and Southeast Asia (Singapore, Thailand, and Indonesia). The countries at the bottom of the ranking were mainly South Asian (Pakistan and Bangladesh) and West Asian (Yemen, Libya, and Syria) countries. The coupling and coordination were generally consistent with the results of the assessment of the three main themes, with higher scores for all three themes leading to a higher degree of coupling and coordination.

### 3.3 Spatiotemporal comparison of theme and sub-theme scores across selected countries in different regions

The global spatial differentiation of CSDI in countries along the MSR was examined. Based on the scores of the CSDI, Moran’s I index was obtained using the global spatial autocorrelation method. In addition, the resulting robustness improved using 999 randomization operations. The results indicated that the global Moran’s I index in 2010, 2015, and 2020 was 0.249, 0.256, and 0.254, respectively, and the null hypothesis was rejected at a significance level of 1%, which meant that the CSDI had a strong spatial dependence. We then selected the countries with the highest and lowest CSDI in each of the five regions based on the aggregation: Europe (Portugal and Bulgaria), Africa (Yemen and Egypt), West Asia (United Arab Emirates and Iraq), South Asia (India and Pakistan) and Southeast Asia (Singapore and Indonesia), and mapped their scores on the social, economic themes and natural sub-themes to further identify the strengths and weaknesses of each region in terms of coastal sustainable development.

The results of theme and sub-theme scores in Europe are shown in Figure S1(a). Portugal was ranked highest in coastal sustainable development for 2010, 2015, and 2020, displaying a good balance and a high degree of growth in the three major themes, as well as better performance in the natural sub-themes. The sea was the foundation and source of Portuguese national identity. Portugal exhibited the largest change in the water sub-theme, particularly in the indicators of clean seawater from 2015 (indicator score = 52.1) to 2020 (indicator score = 75.9), which was related to the creation of the Ministry of the Sea in 2015. Bulgaria was one of the European countries with a lower CSDI score, ranking 33rd, 27th, and 25th in 2010, 2015, and 2020, respectively, and had greatly improved in the last decade. However, its economic and social scores remained poor, and its biodiversity score decreased significantly in 2020.

The results of theme and sub-theme scores in Africa are shown in Figure S1(b), and the study selected the highest-ranked country (Egypt) and the lowest-ranked country (Yemen) for analysis. Yemen performed poorly in coastal sustainable development, ranking 40th, 38th, and 40th in 2010, 2015, and 2020, respectively. Even though its poor economic situation limited its ability to achieve CSDI, it did have certain benefits related to the sub-theme of nature, particularly rich biodiversity. Egypt ranked one of the highest among African countries, ranking 24th, 28th, and 26th in 2010, 2015, and 2020, respectively. It secured high scores in the environment of the atmosphere and coast, and there was a significant increase in economic and water scores. In summary, the level of coastal sustainable development in African countries had experienced more economic and social constraints.

The results of theme and sub-theme scores in West Asia are shown in Figure S1(c). The study selected the highest-ranked country (United Arab Emirates) and typical country (Iraq) affected by conflict in this region for analysis. The United Arab Emirates was the country with the highest level of coastal sustainability in West Asia, ranking 5th, 7th, and 7th in 2010, 2015, and 2020, respectively, and its strengths in the natural sub-themes were mainly in the conservation of biodiversity and the water environment, while its weaknesses were in the atmosphere and land, which were still at a low level (<50). Iraq ranked toward the bottom of the rankings, with the lowest score for sustainable coastal development. It had superior natural conditions but lower economic and social ratings due to the consequences of conflict but quicker development in all themes from 2010 to 2020, with the water environment showing the greatest increase.

The results in South Asia are shown in Figure S1(d). The study selected the highest (India) and lowest (Pakistan) ranked countries affected by the religious culture in this region for analysis. India was ranked relatively high at 25th, 30th, and 31st in 2010, 2015, and 2020, with the main disadvantage being the incongruity between social and economic sustainability and the disadvantage in the nature sub-themes, such as water environment and land resources management. Pakistan lagged, largely at the bottom of the rankings, with disadvantages in the nature sub-theme mainly in biodiversity conservation and coastal protection. The results in Southeast Asia are shown in Figure S1(e). Southeast Asian countries were more internally differentiated, with Singapore scoring better on economic and social sustainability and lower on the nature sub-theme than other countries with higher CSDI indices, while Indonesia was the exact reverse.

In summary, the sub-theme scores of the selected countries showed that South Asia and Africa were characterized by low overall CSDI scores and relatively low sub-theme scores; Southeast Asia and West Asia were significantly disadvantaged in the natural sub-theme; and the majority of European nations had more balanced development with high overall CSDI and sub-theme scores.

## 4 Discussion

### 4.1 Three different levels of coastal sustainable development in different themes

This research developed an integrated CSDI with three dimensions (natural, economic, and social) and highlighted the complexity of coastal ecosystems [55]. According to the overall evaluation results, the factors that caused the difference in the level of sustainable coastal development were mainly social and economic. Therefore, based on the sub-theme scores of selected countries and the future development direction, countries along the MSR could be divided into three development patterns i.e., favorable stage, transitional stage, and unfavorable stage [56,[57], [58]]. And the favorable stage was defined as the one in which all natural, economic and social themes scores were higher than the average of the MSR, the transitional stage was defined as the one in which one of the scores was higher than the average, and the unfavorable stage was defined as the one in which all scores were lower than the average (Fig. 8).

**Fig. 7:**
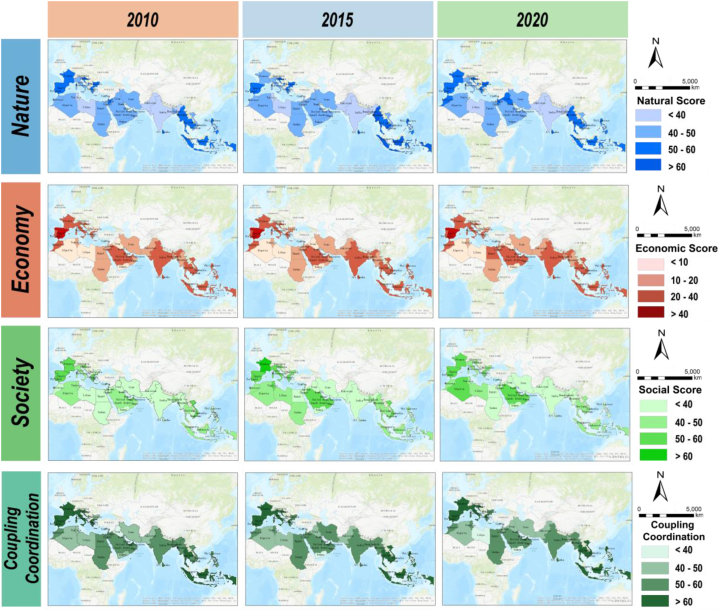
Spatial-temporal distribution of the sustainable development index scores for 2010, 2015, and 2020 at the natural, economic, social, and coupling coordination levels.

**Fig. 8:**
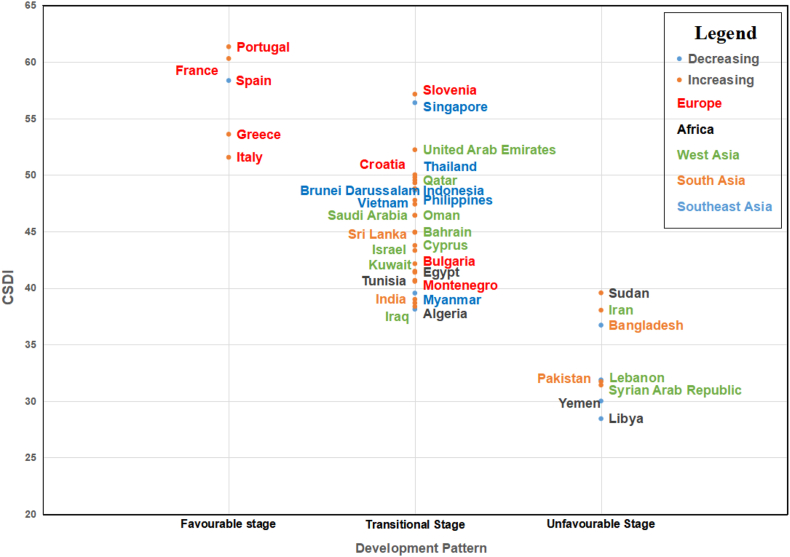
The development pattern, CSDI in 2020, and the changing trends from 2010 to 2020 of the countries along the MSR. The different colored fonts in the graph represent the region where the country is located, and the different colored scatter points represent the changing trend from 2010 to 2020.

The first pattern (favorable stage) included countries with a higher level of natural, economic, and social sustainable development than the overall MSR average, mainly located in Central and Western Europe, which tended to invest more in scientific research and education by implementing innovative development strategies and nurturing international talent. They were often at the forefront of high-tech industrial development and social progress. The major challenge for these countries to further improve the level of coastal sustainable development was to acquire the necessary scientific knowledge regarding the elements that determine the status of the marine environment. The most important of these elements was assessed to the extent possible by quantifying the trade-offs between its various uses based on management decisions [27]. This process necessitated merging the vastly divergent perspectives toward coastal and marine ecosystems and determining the necessary currencies to enable measurement and comparison of quite disparate ecosystem services (e.g., cultural values vs. seafood) [59].

The second pattern (transitional stage) included countries with moderate coastal sustainable development levels and long-term poor performance of a theme dimension or sub-theme dimensions, such as Singapore, Indonesia, Qatar, and the United Arab Emirates (UAE). As shown in the three themes and coupling coordination, most of these countries had higher scores for the economic theme than for the natural theme. As the most typical country, the UAE’s rank for the natural theme lagged significantly behind for the economic and social themes. The UAE is located within the Arabian Gulf and is one of the most affluent per capita nations in the world [60]. Its abundance of oil and gas has motivated a significant push for consumption-based economies. However, with the rapid rise in population in coastal cities, extensive coastal development has increased pressure on coastal ecosystems, leading to the degradation of associated habitats and loss of biodiversity and ecosystem services, resulting in a significant decline in the UAE's biodiversity score in 2015. It is worth recognizing that 15% of its coastal areas and marine waters are established marine protected areas (MPAs). These MPAs protect nearly 12% of the UAE's Exclusive Economic Zone (EEZ), exceeding the 10% conservation target set by SDG14 [61]. However, according to the results of [62] only 10% of the UAE's mangroves were covered by MPAs, and the natural lagoons where mangroves were abundant were rarely left in pristine condition or undamaged. It is important to recognize the need for broader marine area management rather than focusing on MPAs alone. Therefore, these countries should promote the development of shortcomings in the coastal natural environment while ensuring a more comprehensive and detailed development of the advantage themes.

The third pattern (unfavorable stage) included countries with a lower level of natural, economic, and social sustainable development than the overall MSR average, such as Pakistan, Syria, Bangladesh, and Lebanon. Pakistan is a lower-middle-income country, with less than a 3% economic growth rate [63], which has more room for improvement in all theme scores. Pakistan's coastal and marine waters are rich in biological resources and contain a variety of mineral resources. However, several environmental problems were evident along the coast of Pakistan (Karachi and Gadani coasts), such as ship exhaust, oil spills, air pollution, noise pollution, and biological habitat loss. The main sources of pollution were oil refineries, commercial industries, power plants, shipbreaking industries, and ship traffic. There is an urgent need to value coastal sustainable development and further comprehensive integration into the MSR, especially the China-Pakistan Economic Corridor, with its existing development strategies, plans, and policies. Therefore, sectors and actors in these countries should work together across the social, economic, and environmental dimensions of sustainable coastal development while formulating development policies to promote sustainable and comprehensive coastal development.

### 4.2 The uniqueness of coastal ecosystems requires more refined global indicators and assessments

The 2030 Agenda for Sustainable Development incorporates the use and conservation of the ocean and its resources, particularly in coastal areas, within the larger context of sustainable development for the first time [64]. The SDG14, with its seven targets and three definitions of implementation, aims to urgently transform human behavior into sustainable practices in the exploitation of ocean resources and take action to protect productive and resilient oceans [65]. This study examined, to some extent, the development of countries along the MSR to advance SDG14 and draw their attention to coastal ecosystem protection. As shown in Fig. 7, almost all countries have made significant improvements in marine water quality and marine protected areas. However, the slow growth in other scores has placed greater demands on SDG14, particularly in terms of biodiversity. Previous attempts toward SDG14 have concentrated on a limited definition of biodiversity, excluding other crucial aspects, including functional roles, the evolutionary history of species, and unique community assemblages. Countries such as the UAE have accrued far more protected areas than SDG14 requires, but their biodiversity is still a major challenge. The indicators required by SDG14.2 and SDG14.5 were much less precise than what is needed for the coastal zone to safeguard ecological functions, conserve species and habitats, or sustain the delivery of ecosystem services [66], all of which must be evaluated through appropriate indicators and defined thresholds [67].

It is important to note that, according to the results of our study, socioeconomic themes accounted for a greater portion of the variation in CSDI than natural themes, indirectly showing that there is a high level of functional and spatial connectivity within marine ecosystems that is not limited by geographical or administrative boundaries [68]. These situations all called for global indicators and assessment. According to Rees et al. (2018) [53], indicators led by area-based goals alone are insufficient to safeguard the important ecosystem processes and services supported by marine ecosystems. The assessment of the global ocean and coast is also prompted by a variety of expanding ocean concerns that are intimately related to sustainable human development, such as large-scale coral bleaching events [69,70]. A globally coordinated effort based on global indicators and assessment can be nearly twice as efficient as uncoordinated, national-level conservation planning [71]. Big Data for the Planet [72] and The Partnership for Observation of the Global Oceans (POGO) (http://www.ocean-partners.org/training-education) can also provide new technical tools for the refinement of global coastal indicators [73].

## 5 Conclusion

This study fully accounts for the complexity of interactions between coastal ecosystems and human activities and proposes an evaluation system applicable to the assessment of coastal sustainable development levels based on the coupling of the natural-economic-social (N-E-S) ecosystem. The study revealed the level of coastal natural, economic, and social sustainable development of the countries along the Maritime Silk Road (MSR) from 2010 to 2020. Our results showed that the overall level of coastal sustainable development showed a decreasing trend between 2010 and 2015 and a rapid increase between 2015 and 2020. The level of sustainable coastal development varied widely in space, with greater levels in Europe and Southeast Asia and lower levels in South and West Asia and North Africa, which were mainly due to disparities in economic and social development.

Combining the natural, economic, and social differences of countries, the study divided development patterns into three types; it is recommended that the assessment and management of advantages be improved based on compensating for the shortcomings. Based on the findings of the assessment and in the context of SDG14, the study proposed that the borderless nature of coastal ecosystems required a more refined global indicator and assessment. Therefore, for the future, a more comprehensive global coastal zone assessment is necessary. In general, as an indicator system approach, it is difficult to avoid some uncertainty issues when applied to a complex ecosystem such as the coast. Coastal areas are affected by both land and sea, and it is difficult to find comprehensive indicators related to sustainable coastal development when selecting impact factors. However, with the increasing theoretical and methodological integration of SDGs and Earth observation systems, these problems can be greatly improved.

## Author contribution statement

Jian Zuo: Conceived and designed the experiments; Performed the experiments; Analyzed and interpreted the data; Contributed reagents, materials, analysis tools or data; Wrote the paper.

Li Zhang: Conceived and designed the experiments; Analyzed and interpreted the data; Wrote the paper.

Bowei Chen: Conceived and designed the experiments; Performed the experiments; Wrote the paper.

Jingjuan Liao: Analyzed and interpreted the data; Contributed reagents, materials, analysis tools or data.

Mazlan Hashim:Dewayany Sutrisno: Dalhatu Aliyu Sani: Contributed reagents, materials, analysis tools or data; Wrote the paper.

Mohammad Emran Hasan: Riffat Mahmood: Analyzed and interpreted the data; Wrote the paper.

## Additional information

Supplementary content related to this article has been publish online at [URL].

## Data availability

The data that support the findings of this study are openly available in Gitub at https://github.com/zuo-maker/CSDI.

## Declaration of competing interest

The authors declare that they have no known competing financial interests or personal relationships that could have appeared to influence the work reported in this paper.
