# Supplementary material for: Assessment of coastal sustainable development along the maritime silk road using an integrated natural-economic-social (NES) ecosystem
Source: Heliyon. 2023 Jun 19;9(6):e17440. doi: 10.1016/j.heliyon.2023.e17440 (PMC10329136; doi:10.1016/j.heliyon.2023.e17440)
Supplement: Multimedia component 1 [file mmc1.docx]

**Assessment of coastal sustainable development along the Maritime Silk Road through using an integrated natural-economic-social (NES) ecosystem**

*Supplementary Material*

# Text S1. Natural-Economic-Social (NES) complex ecosystem

Ma & Wang (1984) proposed the “Natural-Economic-Social ” complex ecosystem based on the ecological regulation theory of holistic, coordinated, cyclic, and autopoietic. This theory is highly compatible with the three dimensions of sustainable development and is soon applied in sustainable development assessment studies. The coastal ecosystem is a complex symbiosis of social, economic, and ecological systems (Tang et al., 2022). As a theoretical system for studying the internal components of complex systems and their interactions (Goodland & Daly, 1996), the NES complex ecosystem is an important guide for coastal sustainable development assessment studies (Hossain et al., 2020).

# Text S2. **Data Processing**

**Treatment of missing values**: To cope with missing data on statistical indicators for countries at a given time, this study mostly used data from the last year as a proxy (Cheng & Ge, 2020). In response to the lack of data on statistical indicators in some countries in the last 10 years, this study utilized the average of the remaining countries in the study area with data as a replacement.

**Normalization**: Due to the indicator's differences in terms of data features, order of magnitude, and size, this study used the ‘maximum-minimum approach’ to normalize the data to simplify aggregation and comparison across indicators. The indicators are first classified into positive indicators (the larger the value, the more favorable to sustainable development) and negative indicators (the smaller the value, the more favorable to sustainable development) according to the data characteristics. The standardization formulae are Eqs. (1) and Eqs. (2):

| Positive indicators: | $Y_{ij}=\frac{X_{ij}-X_{jmin}}{X_{jmax}-X_{jmin}}$ | (1) |
| --- | --- | --- |
| Negative indicators: | $Y_{ij}=\frac{X_{jmax}-X_{ij}}{X_{jmax}-X_{jmin}}$ | (2) |

$Y_{ij}$is the standardized value; $X_{ij}$ is the initial value of indicator j for country i; and $X_{jmax}$ and $X_{jmin}$ are the maximum and minimum values of indicator j, respectively.

**Weights**: Weights are a quantifiable approach to assess the importance of an indicator in relation to other indicators in the evaluation (Zhang et al., 2006). The entropy weighting model, an objective assignment method (Yan et al., 2014), was utilized in this study. The study assigned the same weights to each of the three themes and five subthemes because they are equally important for coastal sustainable development (Zhang et al., 2022). Therefore, in the evaluation of the subthemes under the nature theme as well as the economic and social themes, the study used the entropy weighting method (Equations (3), (4), (5), and (6)) to determine the weights of the indicators and finally combined the results of the three subtheme scores to achieve the final sustainable development evaluation results.

| $p_{ij}=\frac{Y_{ij}}{\sum_{i=1}^{n} Y_{ij}}$ | (3) |
| --- | --- |
| $e_{j}=-k\sum_{i=1}^{n} p_{ij}\ln(p_{ij})$ | (4) |
| $d_{j}=1-e_{j}$ | (5) |
| $\omega_{j}=\frac{d_{j}}{\sum_{j=1}^{m} d_{j}}$ | (6) |

$Y_{ij}$ is the data value of indicator j for country i after normalization. $p_{ij}$ is the proportion of $Y_{ij}$ in indicator j. $e_{j}$ is the entropy of indicator j. $d_{j}$ is the information utility value of indicator j, and $\omega_{j}$ is the weight of indicator *j*.

# Text S3. **Coupling and Coordination Model**

Based on the capacity coupling system model in physics (Solymar et al., 1996), the coupling and coordination model (CCM) is used to describe the N-E-S system dependency and constraints. The study established CCM to explore the relationship between the three components of sustainable coastal development: environmental, social, and economic.

The coupling degree is calculated using the following formula:

| $C=\sqrt[3]{\frac{N*E*S}{{(N+E+S)}^{3}}}$ | (7) |
| --- | --- |
| $N,E,S=\sum\omega_{subtheme}*\sum(Y_{ij}*\omega_{j})$ | (8) |

where N represents natural sustainability, E represents economic sustainability, S represents social sustainability, $\omega_{\mathrm{subtheme}}$represents the entropy of the natural, economic, and social subthemes, and thus C is the coupling degree between natural, economic, and social sustainability, with a range of [0,1]. The larger the value of C is, the better the natural, economic, and social sustainable development.

Then, the coupling coordination model is introduced to reflect the coordination relationship between complex ecosystems and sustainable development. The formula is:

| $D=\sqrt{C*(\alpha*N+\beta*E+\gamma*S)}$ | (9) |
| --- | --- |

where $\alpha$ is the weight coefficient of natural sustainability, $\beta$ is the weight coefficient of economic sustainability, and $\gamma$ is the weight coefficient of social sustainability. Considering that sustainable development is equally important to the coordination of the three themes, the study defined the same weight of the three ($\alpha$ = $\beta$ = $\gamma$ = 1/3). D represents the coupling coordination degree, and the higher the value is, the higher the level of coordination development among the three.

**Coastal Sustainable Development Index (CSDI):** As shown in Formula (10), the CSDI was constructed by combining the sustainability of the three main themes and their coupled coordination. The study eventually scaled the score to between 0 and 100, with 0 representing the lowest sustainable development level and 100 representing the highest:

| $CSDI=\sqrt{D*(N+E+S)/3}$ | (10) |
| --- | --- |

where CSDI represents the coastal sustainable development index; the higher the value is, the higher the level of coastal sustainable development.

# Text S4. Highlights

1. A natural-economic-social (N-E-S) evaluation system for assessing coastal sustainable development has been established.
2. The level of coastal sustainable development has obvious spatial differences due to the combined effects of economic and social factors.
3. Coastal development patterns were classified into three stages, that is favorable, transitional, and unfavorable).
4. Emphasizing the coastal sustainable development assessment required a more refined global indicator in the context of the 2030 Agenda for Sustainable Development.

# Table S1. Indicators for evaluating the coastal sustainable development level based on the N-E-S ecosystem (UN, 2007; Halpern, 2012; FISO, 2021)

| **Coastal Sustainable Development Index** | **Themes** | **Subthemes** | **Indicators** | **Sources** | **Weight** |
| --- | --- | --- | --- | --- | --- |
|  | Natural Themes | Atmosphere | PM2.5** | Socioeconomic Data and Applications Center (SEDAC) (Hammer et al., 2020) | 0.5998 |
|  |  |  | CO2 emissions per capita* | World Bank Database (2021) | 0.2671 |
|  |  |  | Nitrogen monoxide emissions* | World Bank Database (2021) | 0.1331 |
|  |  | Coast | The proportion of artificial shoreline** | CASEarth Databank (Zhang, 2019) | )0.1572 |
|  |  |  | The proportion of biomass shoreline** | CASEarth Databank (Zhang, 2019) | 0.2895 |
|  |  |  | Coastal protection** | Ocean Health Index (Halpern, 2012) | 0.5533 |
|  |  | Water | Renewable inland water resources per capita* | World Bank Database (2021) | 0.3884 |
|  |  |  | Clean seawater** | Ocean Health Index (Halpern, 2012) | 0.6116 |
|  |  | Biodiversity | Biodiversity** | Ocean Health Index (Halpern, 2012) | 0.6236 |
|  |  |  | Percentage of marine protected area* | World Bank Database (2021) | 0.3764 |
|  |  | Land | The intensity of coastal land development** | ESA Climate Change Initiative-Land Cover led by UCLouvain (2020) | 0.0281 |
|  |  |  | Percentage of coastal forests** | ESA Climate Change Initiative-Land Cover led by UCLouvain (2020) | 0.6744 |
|  |  |  | Percentage of coastal farmland** | ESA Climate Change Initiative-Land Cover led by UCLouvain (2020) | 0.2524 |
|  |  |  | Fertilizer consumption* | World Bank Database (2021) | 0.0451 |
|  | Economic Themes | Infrastructure | Container Terminal Throughput* | World Bank Database (2021) | 0.1229 |
|  |  |  | Liner Shipping Related Index* | World Bank Database (2021) | 0.1574 |
|  |  | Energy consumption | Share of electricity generated from renewable sources* | World Bank Database (2021) | 0.1298 |
|  |  |  | Carbon dioxide emission intensity* | World Bank Database (2021) | 0.1358 |
|  |  | Fisheries | Capture* | Food and Agriculture Organization of the United Nations (2021) | 0.022 |
|  |  |  | Aquaculture* | Food and Agriculture Organization of the United Nations (2021) | 0.0278 |
|  |  | Economic development | GDP per capita* | World Bank Database (2021) | 0.2518 |
|  |  |  | GDP per capita growth rate* | World Bank Database (2021) | 0.0121 |
|  |  |  | Tourism** | Ocean Health Index (Halpern, 2012) | 0.1404 |
|  | Social Themes | Population | Coastal zone population density** | Socioeconomic Data and Applications Center (SEDAC) (CIESIN, 2018) | 0.0206 |
|  |  |  | Population growth rate* | World Bank Database (2021) | 0.1774 |
|  |  |  | Life expectancy* | World Bank Database (2021) | 0.1163 |
|  |  | Research and Education | Education expenditure as a percentage of GDP* | World Bank Database (2021) | 0.1285 |
|  |  |  | R&D Researchers* | World Bank Database (2021) | 0.3588 |
|  |  | Employment | The proportion of the population employed* | World Bank Database (2021) | 0.1983 |

**Note:** Data Types: * Statistical data, ** Geospatial data.

# Supplementary Figure


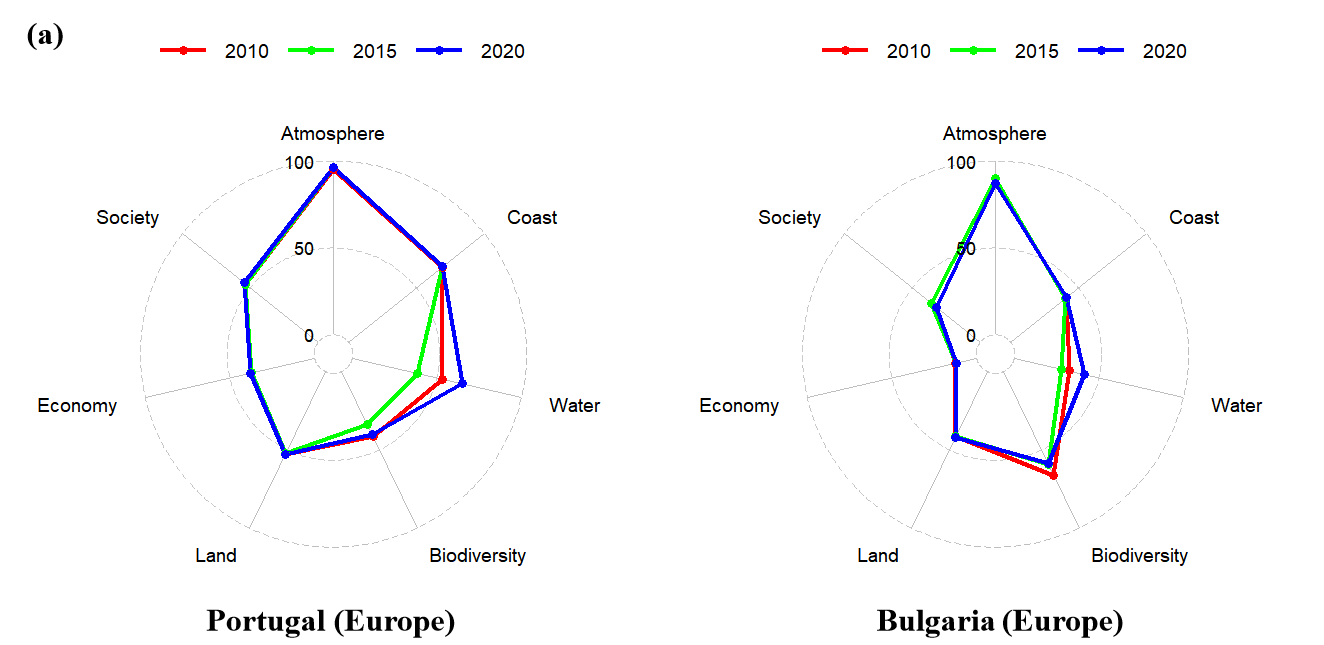


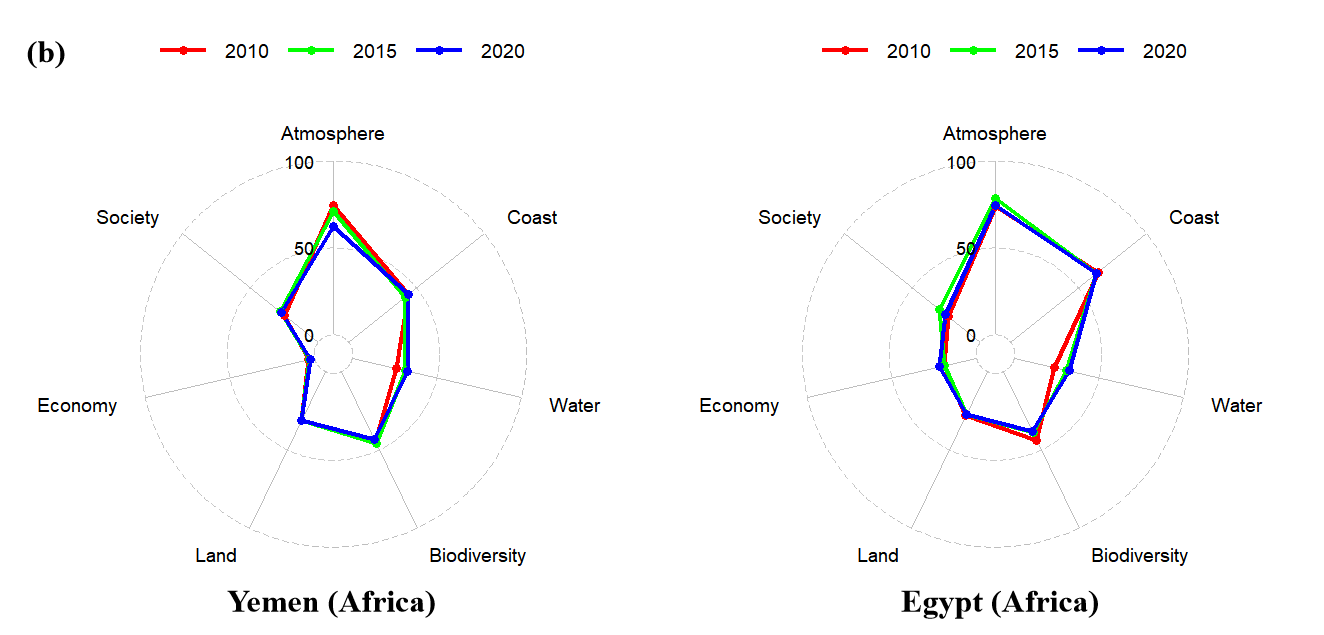


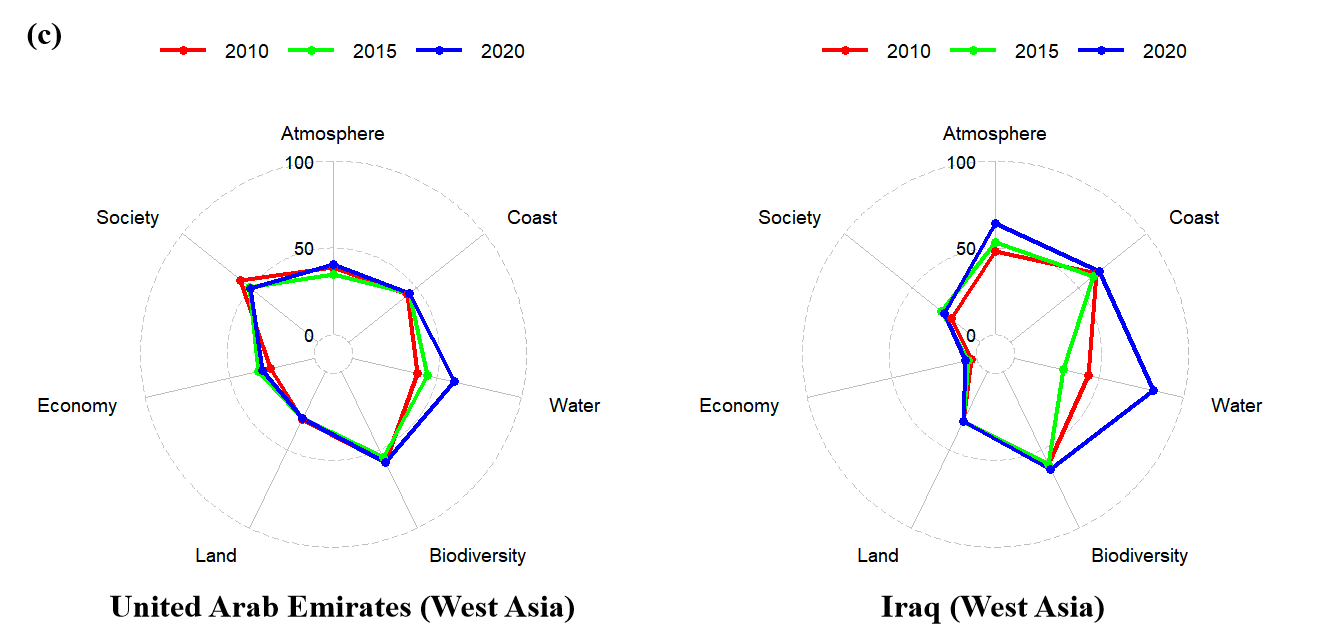


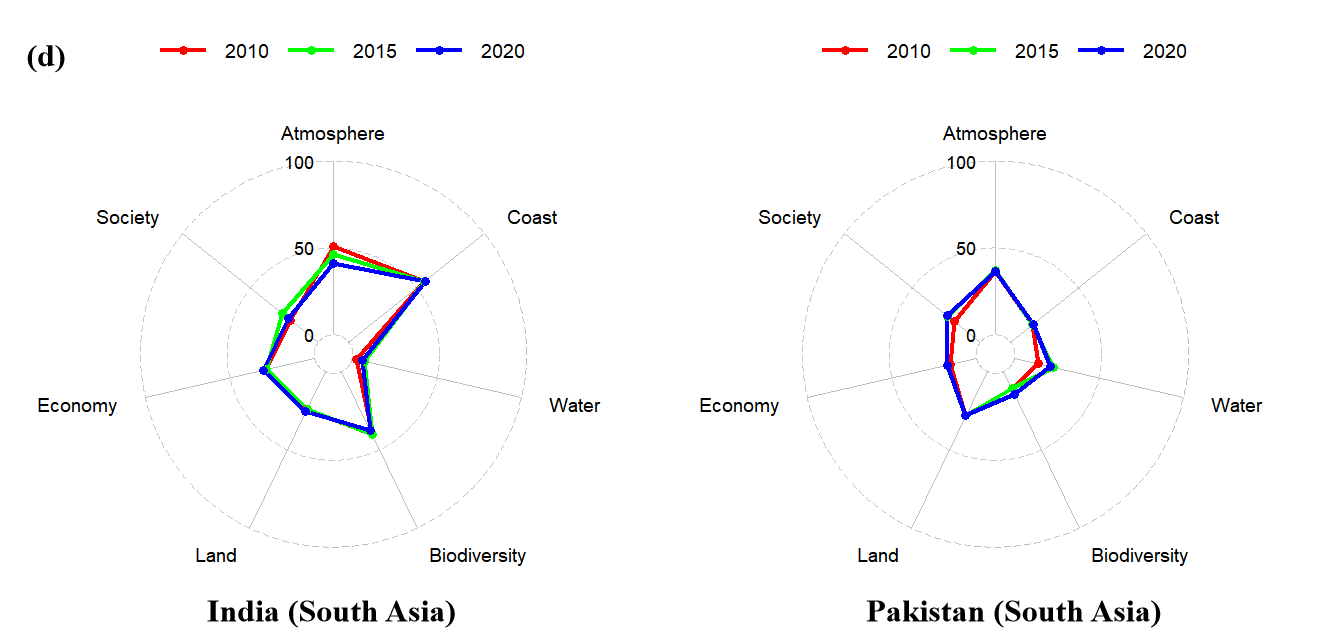

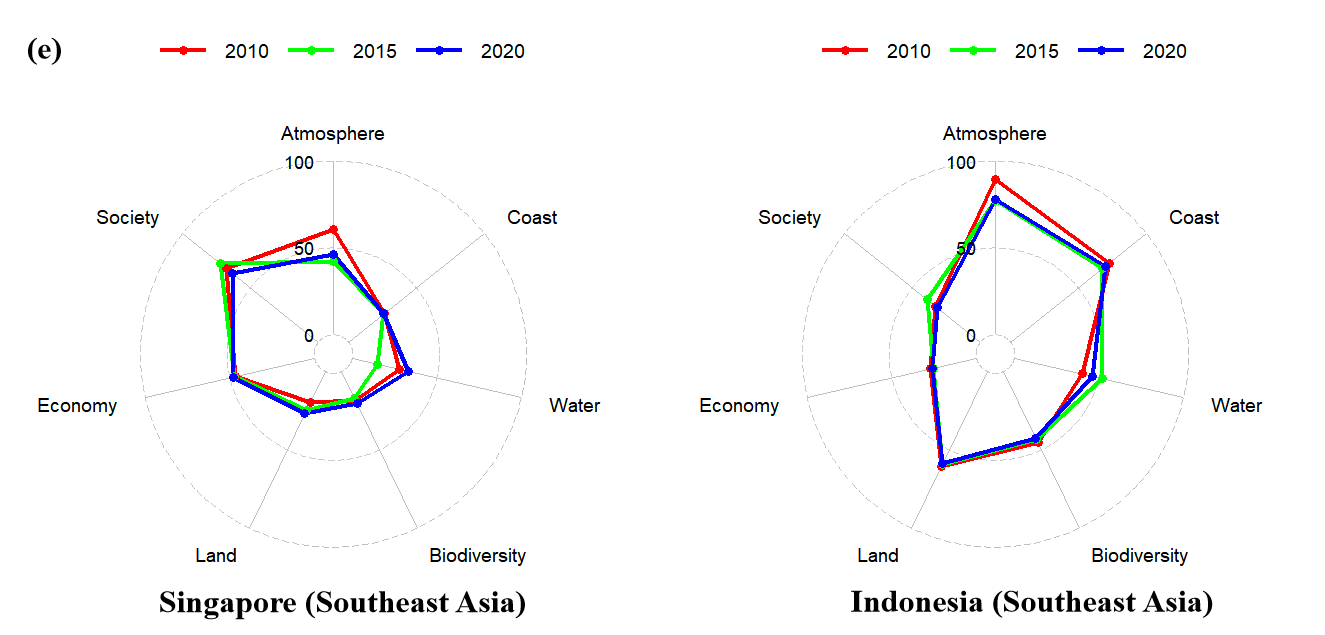


**Figure S1.** The scores of the economic and social themes and the natural subthemes from 2010 to 2020 in countries of (a) Europe, (b) Africa, (c) West Asia, (d) South Asia, and (e) Southeast Asia.


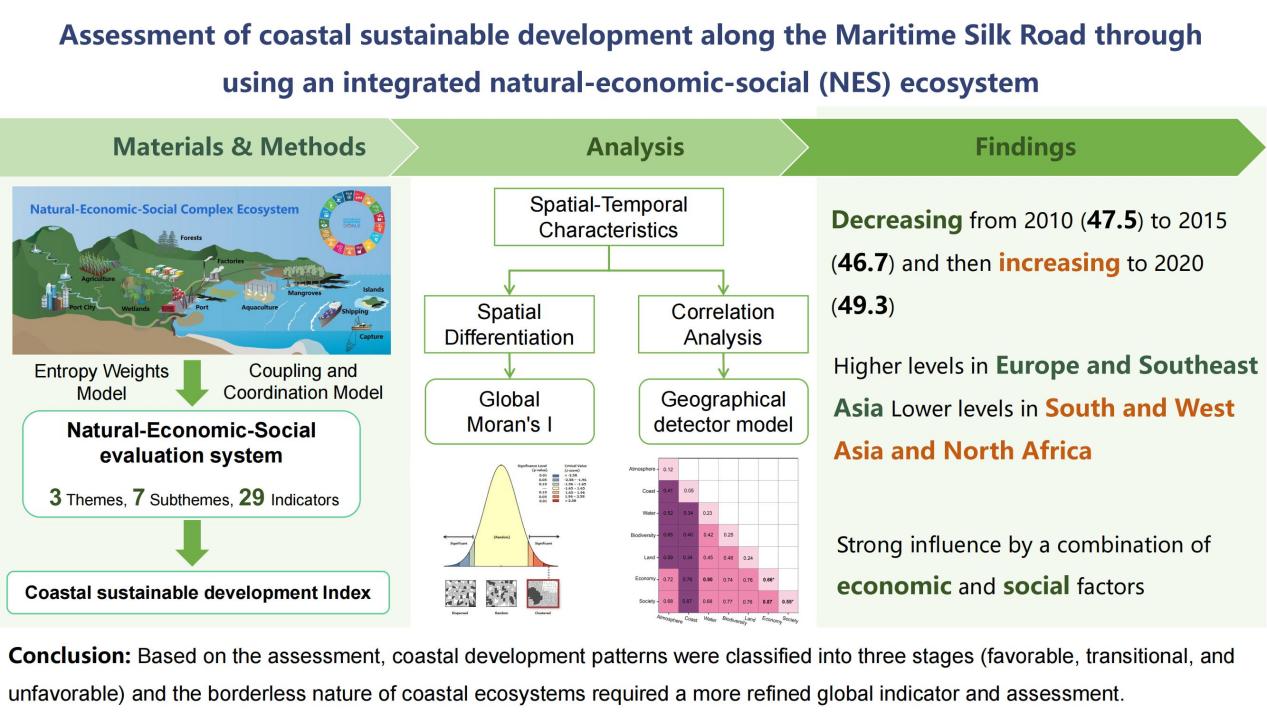


**Figure S2.** Graphical Abstract.

# References

Center for International Earth Science Information Network - CIESIN - Columbia University. 2018. Gridded Population of the World, Version 4 (GPWv4): Population Count, Revision 11. Palisades, New York: NASA Socioeconomic Data and Applications Center (SEDAC). https://doi.org/10.7927/H4JW8BX5. Accessed DAY MONTH YEAR.

Cheng C, Ge C. Green development assessment for countries along the belt and road. Journal of environmental management. 2020 Jun 1;263:110344.

ESCAP U, Scientific C. Integrating the three dimensions of sustainable development: A framework and tools.

Fishery and aquaculture statistics Global production by production source 1950-2016 (FishstatJ), FAO Fisheries and Aquaculture Department [online], Rome ,2018 http://www.fao.org/fishery/statistics/software/fishstatj/en

Fujian Institute For Sustainable Oceans(Xiamen University). Coastal Development Sustainability Report for Countries Along the Maritime Silk Road. 2021.

Halpern BS, Diamond J, Gaines S, Gelcich S, Gleason M, Jennings S, Lester S, Mace A, McCook L, McLeod K, Napoli N. Near-term priorities for the science, policy and practice of Coastal and Marine Spatial Planning (CMSP). Marine Policy. 2012 Jan 1;36(1):198-205.

Halpern BS, Longo C, Hardy D, McLeod KL, Samhouri JF, Katona SK, Kleisner K, Lester SE, O’Leary J, Ranelletti M, Rosenberg AA. An index to assess the health and benefits of the global ocean. Nature. 2012 Aug;488(7413):615-20.

Hammer MS, van Donkelaar A, Li C, Lyapustin A, Sayer AM, Hsu NC, Levy RC, Garay MJ, Kalashnikova OV, Kahn RA, Brauer M. Global estimates and long-term trends of fine particulate matter concentrations (1998–2018). Environmental Science & Technology. 2020 Jun 3;54(13):7879-90.

Solymar L, Webb DJ, Grunnet-Jepsen A. The physics and applications of photorefractive materials. Clarendon Press; 1996 Aug 15.

United Nations. Indicators of Sustainable Development: Guidelines and Methodologies. New York, 2007.

World Bank. World development report 2017: Governance and the law. The World Bank; 2017 Jan 23.

Yan J, Feng C, Li L. Sustainability assessment of machining process based on extension theory and entropy weight approach. The International Journal of Advanced Manufacturing Technology. 2014 Mar;71(5):1419-31.

Zhang J, Wang S, Pradhan P, Zhao W, Fu B. Untangling the interactions among the Sustainable Development Goals in China. Science Bulletin. 2022 May 15;67(9):977-84.

Zhang L. 30m Landsat TM/OLI shoreline variation dataset along the the Maritime Silk Road from 1990 to 2020. Aerospace Information Research Institute, Chinese Academy of Sciences, 2019-10-15.21.86109/casearth.5da578a9329b5613607cc967;http://hdl.pid21.cn/21.86109/casearth.5da578a9329b5613607cc96

Zhang Y, Yang Z, Li W. Analyses of urban ecosystem based on information entropy. Ecological Modelling. 2006 Aug 10;197(1-2):1-2.
